# Safety Evaluation and Biodistribution of Fetal Umbilical Cord Mesenchymal Stem Cells-Derived Small Extracellular Vesicles in Sprague Dawley Rats

**DOI:** 10.3390/ijms26146806

**Published:** 2025-07-16

**Authors:** Illayaraja Krishnan, Ubashini Vijakumaran, Ng Min Hwei, Law Jia Xian, Mohd Rafizul Mohd Yusof, Thavachelvi Thangarajah, Tan Geok Chin, Yin Ping Wong, Anusha Kalyanasundaram, Zalina Mahmood, Shathiya Rajamanickam, Baskar Subramani, Yogeswaran Lokanathan

**Affiliations:** 1 Department of Tissue Engineering and Regenerative Medicine (DTERM), Faculty of Medicine, Universiti Kebangsaan Malaysia (UKM), Cheras, Kuala Lumpur 56000, Malaysia; p114013@siswa.ukm.edu.my (I.K.); ubashinivijakumaran@ukm.edu.my (U.V.);; 2 National Pharmaceutical Regulatory Agency (NPRA), Lot 36, Jalan Prof Diraja Ungku Aziz, Petaling Jaya 46200, Malaysia; 3 Department of Parasitology and Medical Entomology, Faculty of Medicine, Universiti Kebangsaan Malaysia (UKM), Kuala Lumpur 56000, Malaysia; rafizulyusof@hctm.ukm.edu.my; 4 Department of Obstetrics and Gynaecology, Hospital Angkatan Tentera (HAT) Tuanku Mizan, Kuala Lumpur 53300, Malaysia; chelvi777@yahoo.com; 5 Department of Pathology, Faculty of Medicine, Universiti Kebangsaan Malaysia (UKM), Kuala Lumpur 56000, Malaysiaypwong@hctm.ukm.edu.my (Y.P.W.); 6 Department of Pathology, Hospital Raja Permaisuri Bainun (HRPB), Ipoh 30450, Malaysia; anushapathology@gmail.com; 7 Production and Blood Supply Management Division, National Blood Centre, Kuala Lumpur 50400, Malaysia; dr.zalina@moh.gov.my; 8 Medixcell Sdn. Bhd., Level 5, Equatorial Plaza, Lot 5-5 & 5-6, Kuala Lumpur 50250, Malaysia; shakthi_rj21@yahoo.com (S.R.); sudabas23@gmail.com (B.S.); 9 Advance Bioactive Materials-Cells UKM Research Group, Universiti Kebangsaan Malaysia (UKM), Bangi 43600, Malaysia

**Keywords:** umbilical cord, mesenchymal stem cells, small extracellular vesicles, intravenous, safety, biodistribution

## Abstract

Umbilical cord mesenchymal stem cells (UCMSCs)-derived small extracellular vehicles (sEVs) are reported to offer therapeutic effects in regenerative medicine, but they lack safety and biodistribution profiles to support smooth translation at the clinical stage and regulatory requirements. Our study aimed to determine the safety and biodistribution profile in a healthy animal model before application in the metabolic syndrome model. Method: Healthy male Sprague Dawley (SD) rats were given an intravenous (IV) injection of normal saline (control group) or pooled fetal UCMSCs-derived sEVs (treated group) every three weeks for 90 days. Morbidity and mortality observation (daily), physical measurements (weekly), selected serum biochemistry (every three weeks), and hematology (every three weeks) were performed for 90 days. Acute toxicity (on day 14) and sub-chronic toxicity (on day 90) were assessed for gross necropsy, relative organ weight, and histopathological assessment of lungs, liver, spleen, kidney, and lymph nodes. Separately, a biodistribution study was conducted with the sEVs preparations labeled with PKH26 fluorescent dye, given intravenously to the rats. The organs were harvested 24 h post-injection. There were no drastic changes in either group’s morbidity or mortality, physical, hematological, and biochemistry evaluation. The histopathological assessment concluded moderate (focal) inflammation in the treated group’s kidneys and signs of recovery from the inflammation and vascular congestion in the liver. A biodistribution study revealed a higher accumulation of sEVs in the spleen. Multiple IV injections of the pooled fetal UCMSCs-derived sEVs in healthy male SD rats were deemed safe. The sEVs were abundantly distributed in the spleen 24 h post-injection.

## 1. Introduction

Mesenchymal stem cells (MSCs) are multipotent cells that hold promise for regenerative medicine due to their immunomodulatory and regenerative properties. According to the International Society for Cell and Gene Therapy (ISCT), MSCs must meet three criteria: (i) plastic adherence, (ii) trilineage differentiation into osteoblasts, adipocytes, and chondroblasts, (iii) positive expression of CD105, CD73, and CD90 surface markers and lack of CD45, CD34, CD14 or CD11b, CD79 alpha or CD19, and HLA-DR surface molecules [1,2]. Despite being evaluated in clinical trials for over a decade, clinical trials often fail to replicate pre-clinical results [3]. Several factors may contribute to these discrepancies, including the risk of tumorigenesis [4], immune rejection, toxicity [5], and challenges with transport and storage stability [6]. To mitigate these risks, researchers are exploring cell-free therapies. Given that the beneficial effects of MSCs are primarily due to their paracrine activities, evidence increasingly suggests that the biological functions of stem cells are largely due to the extracellular vesicles (EVs) they secrete [7].

In recent years, MSCs-derived EVs have emerged as a safer and more effective treatment in regenerative medicine, surpassing MSCs. Small EVs (sEVs) are nanosphere vesicles typically less than 200 nanometers [8] with a phospholipid bilayer that carries bioactive molecules such as proteins, lipids, and nucleic acids [9]. These nanovesicles have desirable properties, including regeneration, differentiation, angiogenesis, and minimal immunogenicity, which makes the item ideal for nanomedicine applications [10,11,12]. Unlike MSCs, EVs can prevent tumorigenic complications due to their lack of cellular components. Additionally, EVs isolation, storage, and dosage are more precisely defined and controlled than MSCs [13]. Human umbilical cord mesenchymal stem cells extracellular vesicles (UCMSCs)-derived EVs have been studied for various applications, including spinal cord injury recovery by maintaining the blood-spinal cord barrier’s structural integrity and alveolarization in the bronchopulmonary dysplasia rat model [14]. UCMSCs-derived EVs alleviated the autoimmune disorder dacryoadenitis by polarizing macrophages [15] and rheumatoid arthritis by T-lymphocyte immunomodulation [16]. They are also widely used in diabetic applications, including wound healing [17] and diabetic neuropathy [18]. However, the expanding use of UCMSCs-derived EVs across various treatments raises potential safety concerns.

Research studies have indicated that extracellular vesicles (EVs) may carry toxic biomarkers and harmful microRNAs (miRNAs), raising concerns about their safety in clinical applications [19,20]. For instance, intravenous injection of fibroblast-derived EVs has been associated with mild inflammation in the liver and kidney [21]. In contrast, MSC-derived EVs [22], HEK293T-derived EVs [23,24], and adipose stem cells (ASCs)-derived EVs [25] have shown no adverse effects in hematological indices, clinical signs, gross necropsy, or histopathological assessments. HEK293T-derived EVs also exhibited minimal immune activation and no signs of systemic toxicity [24] and showed minimal cytotoxicity in human whole blood assay [26].

Regarding tumorigenicity, the data are similarly mixed. MSC-derived EVs have been shown to promote cancer progression in some cases, particularly bone marrow-derived MSC EVs, which can activate the Hedgehog signaling pathway in osteosarcoma and gastric cancer [27]. Likewise, adipocyte-derived EVs have been reported to promote breast cancer development [28]. However, other studies suggest that MSC-derived EVs can exert anti-tumor effects; for example, they inhibited prostate cancer growth through the delivery of miR-145. These mixed findings emphasize the necessity of conducting rigorous toxicity and safety assessments prior to the clinical application of EVs.

The absence of extensive multi-center trials further limits our understanding of the efficacy and toxicity of EVs in human applications [29]. The complex nature of EVs presents significant challenges due to their largely unknown biological functions, mechanisms of action, and pharmacokinetics [30]. Meanwhile, researchers have also highlighted isolation methods, incomplete characterization, and the targeted site of action as limitations for clinical translation. Safety profile assessments are also insufficient, which raises concerns regarding the potential risks associated with EV therapies [31]. These factors collectively become substantial hurdles for the manufacturing of clinical-grade EVs. Rigorous safety evaluations, including biodistribution and excretion using both in vitro and in vivo models, are essential prerequisites for pharmaceutical products before human clinical trials [32,33,34]. Despite significant findings from preclinical animal studies, there are still a limited number of clinical trials to assess the safety and effectiveness of MSCs-derived EVs. As a result, currently, no MSCs-derived EVs-based products have received regulatory approval or are available on the market [35,36,37].

Recent studies have investigated the biodistribution of EVs upon their administration into recipient animals in vivo, aiming to better understand both on-target and off-target effects [38]. Notably, Grange’s research elucidates target specificity, demonstrating that MSCs-derived EVs are specifically localized in the kidneys of kidney-injured mice compared to control subjects [39]. Studies do report that different cell sources of EVs carry different biodistribution patterns [40,41]. However, the holistic analysis concluded that EVs typically accumulate in specified organs such as the liver, lungs, kidneys, and spleen regardless of cell source and target model [42]. Kang’s systematic review uncovers significant variability in methodology across EV biodistribution studies. This variability encompasses factors such as EV dose, target organ, tracking method (e.g., fluorescent dyes, bioluminescence, radiolabeling), and EV isolation techniques, and the authors emphasized the urgent need for standardized guidelines to enhance the reliability and comparability of EV biodistribution research [42]. Hence, biodistribution evaluation before the administration of EVs in a disease model at a preclinical stage is encouraged.

Thus, we aimed to evaluate the safety profile and biodistribution of sEVs derived from human fetal UCMSCs when administered to healthy male Sprague Dawley (SD) rats. The sEVs were harvested from MSCs grown from the fetal part of the umbilical cord. A series of studies have reported that fetal UCMSCs showed a higher viability and proliferation rate than the maternal part of UCMSCs [43,44]. Fetal UCMSCs also inhibited memory T-cells and posed better immunomodulatory properties than maternal MSCs [45]. Generally, UCMSCs also lack surface levels of human leukocyte antigen (HLA) class I [46,47], thus making them less immunogenic than adult MSCs [48]. To the best of our knowledge, this study elucidated the pooled fetal UCMSCs-derived sEVs’ safety and biodistribution profile after being administered in multiple doses via intravenous (IV) injection in male SD rats for the first time. This investigation aims to systematically assess any adverse effects or potential toxicities associated with administering pooled fetal UCMSCs-derived sEVs intravenously with multiple doses. The study encompasses a comprehensive evaluation, including morbidity and mortality observations, physical assessment, hematological, biochemistry, necropsy, and histopathological assessment, and biodistribution to provide a detailed safety profile. Our study presents potential risks and side effects of pooled fetal UCMSCs-derived sEVs early in the pre-clinical development process, allowing for the refinement of the therapy before it is tested in the metabolic syndrome disease model.

## 2. Results

### 2.1. Physical Measurements, Observation, Selected Serum Biochemistry, and Full Blood Count

**Figure 1:**
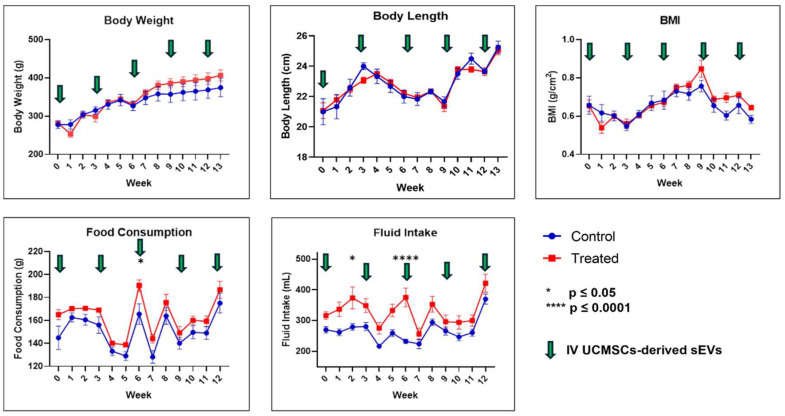
Physical measurements of body weight, body length, body mass index, food consumption, and fluid intake. Data were presented as mean ± SEM (*n* = 6 rats per group) for the control and treated groups at weeks 0, 3, 6, 9, and 13. A difference at *p* ≤ 0.05 was considered statistically significant.

As presented in Figure 1, throughout the study, it was observed that BW, BL, and BMI were not statistically significantly different between the control and treated groups at any time point. Besides that, food consumption at week 6 (*p* ≤ 0.05) and fluid intake at weeks 2 (*p* ≤ 0.05) and 6 (*p* ≤ 0.0001) showed statistically significant differences, whereby they are higher in the treated group. No death occurred in either group, and there was an absence of morbidity and mortality signs and symptoms as described in Supplementary Table S1 from the acclimatisation period to the end of the study.

**Figure 2:**
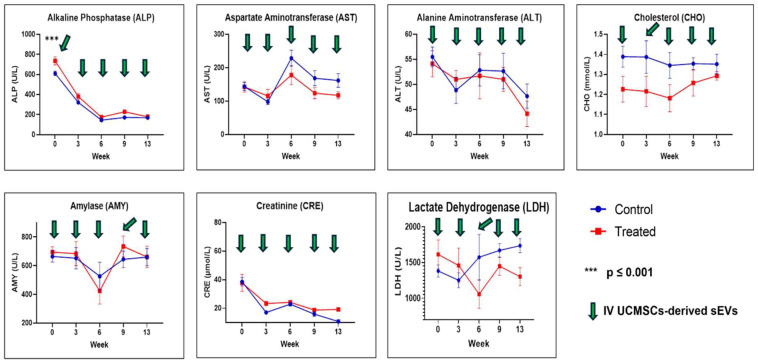
Selected serum biochemistry of ALP, AST, ALT, CHO, AMY, CRE, and LDH. Data were presented as mean ± SEM (*n* = 6 rats per group) for the control and treated groups at weeks 0, 3, 6, 9, and 13. A difference at *p* ≤ 0.05 was considered statistically significant.

For the selected serum biochemistry results, as illustrated in Figure 2, all parameters were not statistically significant between both groups except for ALP at week 0 (*p* ≤ 0.001); the treated group had a higher level than the control group.

**Figure 3:**
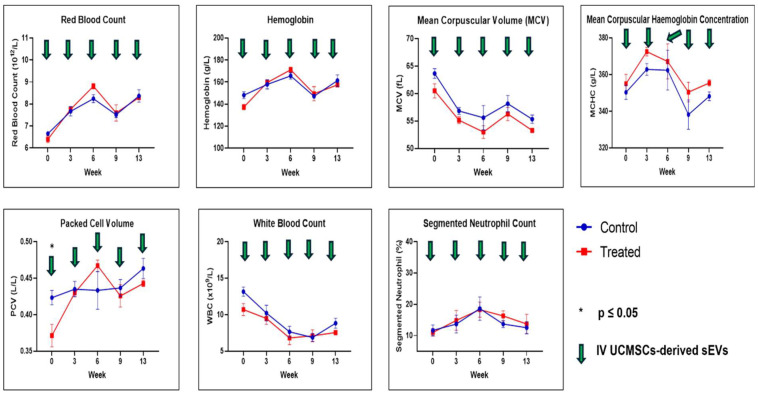
Full blood count of RBC, Hb, MCV, MCHC, PCV, WBC, and Segmented NEUTRO. Data were presented as mean ± SEM (n = 6 rats per group) for the control and treated groups at 0, 3, 6, 9, and 13 weeks. A difference at *p* ≤ 0.05 was considered statistically significant.

**Figure 4:**
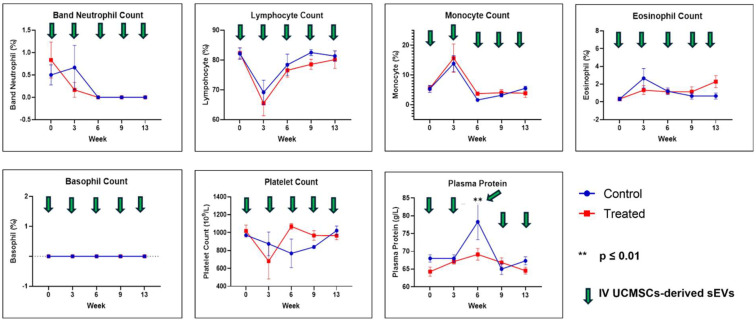
Full blood count of Band NEUTRO, LYMPH, MONO, EOSIN, BASO, PLT, and plasma protein. Data were presented as mean ± SEM (n = 6 rats per group) for the control and treated groups at weeks 0, 3, 6, 9, and 13. A difference at *p* ≤ 0.05 was considered statistically significant.

FBC analysis results are shown in Figure 3 and Figure 4. All the FBC parameters were not statistically significant except for PCV and plasma protein results between both groups. For PCV, the results were statistically significantly different (*p* ≤ 0.05) at week 0, and for plasma protein, the level was statistically significant (*p* ≤ 0.01) at week 6 between both groups. In both cases, their levels were higher in the control group compared to the treated group.

### 2.2. Gross Necropsy Evaluation, Relative Organ Weight, and Histopathology Assessment

**Figure 5:**
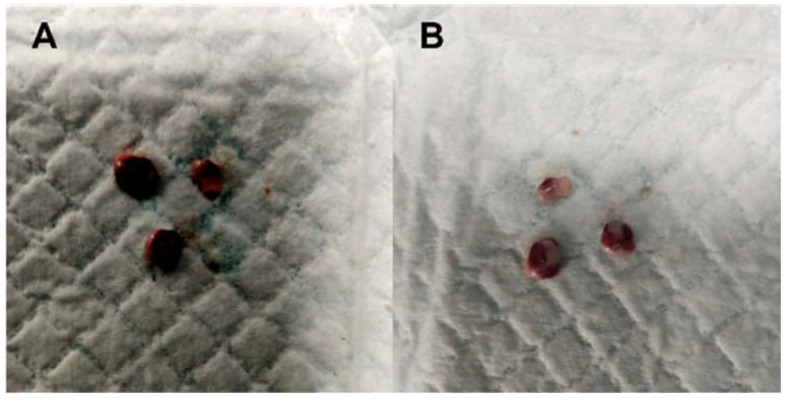
Gross necropsy of the lymph nodes at day 90 (sub-chronic toxicity). (**A**) control group (*n* = 6) with dark brown appearance and (**B**) treated group (n = 3 from n = 6) with reddish appearance.

As shown in Table 1, gross necropsy evaluation of the harvested organs at Day 14 (acute toxicity) showed no physical appearance abnormalities. Lymph nodes were not assessed for acute toxicity. The same observation was concluded for both the lungs and kidneys of the rats for both groups at day 90 (sub-chronic toxicity). The mottled edge appearance of the liver and the blunt edge appearance of the spleen were observed for both groups, with an equal number of rats for each group at day 90. Meanwhile, for the lymph nodes, only the treated group showed a reddish appearance (n = 3), and this finding was absent in the control group with the usual dark brown appearance, as illustrated in Figure 5.

**Figure 6:**
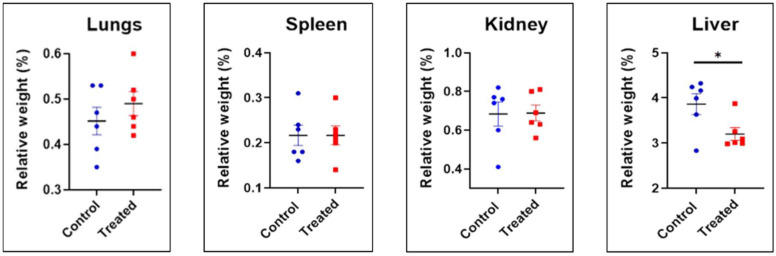
Relative organ weight (%) of lungs, spleen, kidney, and liver at D90 (sub-chronic toxicity). Data were presented as mean ± SEM (n = 6 rats per group) for the control and treated groups. A difference at * *p* ≤ 0.05 was considered statistically significant.

Based on the relative organ weight (%) of the lungs, kidneys, and spleen, no statistically significant differences were noted compared to the control and the treated groups. In the liver, a statistically significant reduction (*p* ≤ 0.05) in relative liver weight (%) was observed in the treated group, as seen in Figure 6.

**Figure 7:**
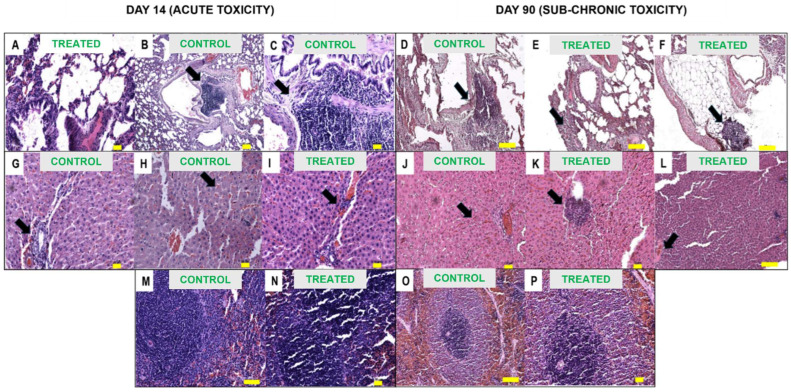

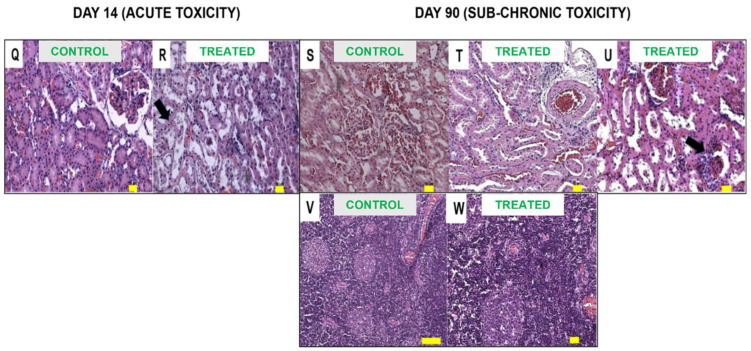
Histopathology analysis. (**A**) Normal lung, (**B**) lung with perivascular inflammation, (**C**) peribronchial inflammation lung, (**D**) lung with perivascular lymphocytic infiltrates region, (**E**) lung with focal and mild lymphoplasmacytic infiltrate at interstitium, (**F**) lung with focal and moderate lymphoplasmacytic infiltrate at interstitium, (**G**) mild to moderate periportal inflammation of liver, (**H**) mild vascular congestion of liver, (**I**) moderate periportal inflammation and vascular congestion of liver, (**J**) liver with moderate vascular congestion, (**K**) liver with focal and mild to moderate periportal lymphocytic infiltrates, (**L**) liver with focal mild vascular congestion and periportal lymphocytic infiltrates, (**M**,**N**) normal lymphoid follicles of spleen, (**O**,**P**) spleen with normal lymphoid follicles, (**Q**) normal kidney, (**R**) mild tubular dilatation of kidney, (**S**) normal kidney, (**T**) kidney with focal mild to moderate lymphocytic infiltrates, (**U**) kidney with mild tubular dilatation, (**V**,**W**) normal lymph nodes. Images were captured with a scale bar of 10 µm and magnification of 10× (**D**–**F**,**L**,**M**,**O**,**V**) and 20× for the rest. Histopathology assessment at day 14 after treatment for the evaluation of acute toxicity (**A**–**C**,**G**–**I**,**M**,**N**,**Q**,**R**). Histopathology assessment at day 90 after treatment for the evaluation of sub-chronic toxicity (**D**–**F**,**J**–**L**,**O**,**P**,**S**–**U**,**V**,**W**).

Figure 7 shows the histopathological micrographs of the harvested organ for both acute and sub-chronic toxicity, and Table 2, Table 3, Table 4, Table 5 and Table 6 summarize the histopathology assessment. Acute toxicity evaluation on Day 14 of the treatment showed mixed results. The spleen showed no abnormalities for both groups. In the lungs, manifestation of severe (focal) lymphocytic infiltrate at the peribronchial region for the control group was noted, and this finding was absent in the treated group. Mild necrosis was observed in both groups for the liver, and mild apoptosis was observed in the treated group only. Mild and mild to moderate lymphocytic inflammatory cell infiltrates were observed in the control and treated groups, respectively. Mild vascular congestion was observed in the control group, and it was moderate in the treated group. Mild tubular changes were observed in the treated kidney, but this finding was not observed in the control group.

In sub-chronic toxicity evaluation at day 90 of the treatment, the spleen for both groups was found to be healthy without any abnormalities. In the case of lymph nodes, mild lymphovacular dilatation was noted for both groups, but this could be normal, according to the pathologist. Mild hemorrhage was pointed out in the control group’s lungs and was not observed in the treated group. Severe (focal) lymphocytic infiltrate and peribronchial were observed in the control group, and there was severe (focal) lymphoplasmacytic infiltrate at the interstitium and soft tissue in the lung of the treated group. Nevertheless, both groups were affected in the lungs. In both groups, mild lymphocytic infiltrates were observed in the liver. In addition, mild vascular congestion was observed in the treated group’s liver compared to moderate vascular congestion in the control group. Meanwhile, moderate (focal) lymphocytic infiltrates in the kidney were observed in the treated group compared to the control group. Mild tubular changes in the kidney were observed in both groups.

### 2.3. Biodistribution Study

**Figure 8:**
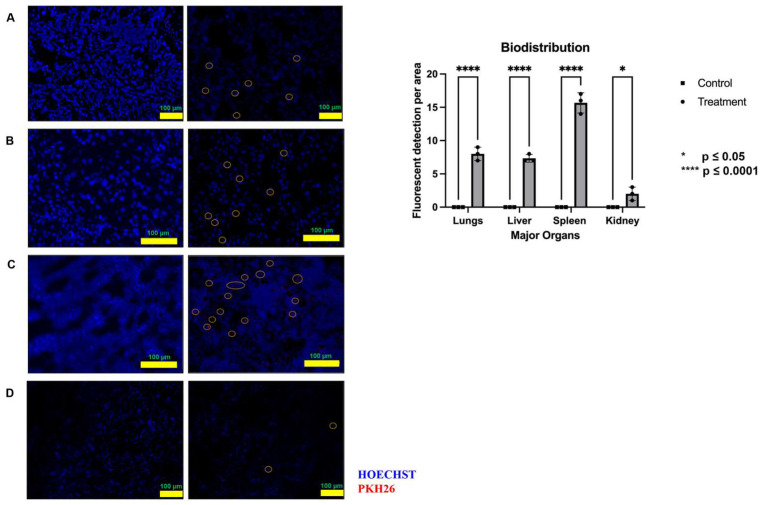
Tracing of PKH26 fluorescent dye-labeled pooled fetal UCMSCS-derived sEVs preparations in the major harvested organ. (**A]** lungs, (**B**) liver, (**C**) spleen, and (**D**) kidney. The left diagrams are control, and the right diagrams are PKH26 dye-labeled sEVs. Images were captured at a magnification of 10× (**A**,**D**) or 20× (**B**,**C**) with a scale bar of 100 µm. The presence of red PKH26 fluorescent dye was circled in the images. n = 3 from the control group (PBS). n = 3 from the treated group (PKH26 labeled sEVs).

Figure 8 shows the images captured to study the biodistribution of pooled fetal UCMSCs-derived sEVs preparations. After 24 h, it was well distributed in most rats’ metabolic organs and detected with red fluorescent images. They were most abundantly biodistributed in the spleen, followed by the lungs and liver, after a 24 h IV injection. The least biodistributed was seen in the rats’ kidneys.

## 3. Discussion

EVs as a potential nanomedicine are developing innovative biomedicines, and for the triumph of translational to clinical, there is a need to address vast areas such as quality, safety, and efficacy due to the complexity and heterogeneity nature of EVs [49]. UCMSCs-derived sEVs preparations as nano biomedicines have been proven to have a high potential for various illnesses such as kidney, liver, ocular, spinal cord, and neurodegenerative ailments [20,50,51]. Most studies focus on the efficacy of the UCMSCs-derived sEVs preparations; an equally critical component, the safety study, has often been neglected in the pre-clinical stages. Given that EVs are bioactive cargo with highly complex contents, they may contain toxic proteins and hazardous miRNAs in addition to biomedicines substances [29]. The safety of EVs is currently unsettled in this bionanotechnology field, especially in clinical research and using large animal models [52]. This aspect is vital for determining the safety of EVs during the pre-clinical drug development stage to assess risks and ensure smooth translation with solid support at the clinical stage [53,54,55]. The primary goal of the clinical trial Phase 1 (volunteers) and 2a (patients) in EVs-based biomedicines is the safety of the recipients. Therefore, extensive product testing is required to evaluate the risk of safety concerns [56]. However, there are no globally recognized guidelines to define EVs and their clinical applications, and there is no harmonized standard for evaluating the safety of EVs at the pre-clinical and clinical stages of drug development [20]. EVs’ toxicity and safety assessment may involve general toxicity, immunogenicity/immunotoxicity, gene toxicity, and tumorigenicity [57], depending on the cell sources and the target disease.

This study revealed that pooled fetal UCMSCs-derived sEVs preparations did not cause any abnormalities in the physical appearances of the healthy male SD rats. Thus, fetal UCMSCs-derived sEVs preparations did not cause physical abnormalities in healthy male SD rats. BW, BL, and BMI are parameters indicating healthy growth patterns. Abnormalities in growth patterns can cause growth hormone imbalances and tumorigenicity [6,58,59,60]. The growth rates for both groups in our study were similar, suggesting nominal effects of pooled fetal UCMSCs-derived sEVs IV injections in the healthy rat model. The treated group did not show any reduction in food or fluid intake compared to the control group throughout the study, indicating that their health was unaffected by the IV administration of the pooled fetal UCMSCs-derived sEVs preparations. Food consumption and fluid consumption were higher trends in the treated groups than in the control group. Reduction in food consumption and drink intake is associated with sickness and toxicology issues after the administration of potential drugs [61]. Generally, in toxicity studies, BW reduction is associated with treatment-induced toxicity due to lower food consumption; however, in this study, no decrease in food consumption occurred [62].

Various morbidity and mortality signs and symptoms, as described in Supplementary Table S1, were used to assess the health status of the healthy male SD rats as in previous research [58,63,64]. This study concluded that all the morbidity and mortality signs and symptoms were absent, as reported by other researchers using EVs via the IV route [21,22,23,24]. This includes the absence of systemic anaphylaxis shock response post-IV injections of pooled fetal UCMSCs-derived sEVs preparations [22].

Hematological assessment and analysis reported no significant differences between the control and treated groups except for PCV and plasma protein. This finding in this study aligns with the previous research [22]. Plasma proteins, mainly albumin and globulin, are synthesized in the liver. In our study, a significant reduction in plasma protein was observed in the treated group compared to the control. This implies that the UCMSCs-derived sEVs preparations caused hypoproteinemia in the treated group. An increase in metabolism and inflammation is associated with reduced protein concentration in blood [65,66]. In the liver, there are signs of recovery of lymphoplasmacytic inflammation in the treated group from mild to moderate (acute) to mild (sub-chronic). Therefore, the reduced plasma proteins were observed in week 6 for the treated group, and thereafter, no significant differences in plasma proteins were observed in both groups. Selected serum biochemistry tests also showed no significant toxicity observed between both groups, except for ALP, with significantly higher (*p* ≤ 0.001) values at week 0 for the treated group before starting the treatment. We could not find a reasonable explanation for this finding since other liver enzyme levels were not showing any differences at week 0 for both groups.

Based on the necropsy evaluation, relative organ weight (%), and histopathology assessment, both the spleen and lymph nodes were not affected in either group, even though mild lymphovacular dilatation was detected in both groups for the lymph nodes. This could be a regular occurrence, according to the histopathologist’s justification. These results indicated that the pooled fetal UCMSCs-derived sEVs preparations do not activate rat immune responses post-IV injection using multiple doses. Moderate (focal) lymphoplasmacytic inflammation was observed in the kidneys of the treated group, and this finding was also observed in another study [21] using a different source of MSCs EVs.

Meanwhile, in the liver, there are signs of recovery of lymphoplasmacytic inflammation in the treated group from mild to moderate (acute) to mild (sub-chronic), and these results suggest that pooled fetal UCMSCs-derived sEVs have a positive outcome of liver inflammation in long-term administration [67]. The same trend was observed for vascular congestion in the hepatocyte. A significant reduction in the relative liver weight (%) was observed in the treated group. However, based on the gross necropsy evaluation and histopathology assessment, there is no association with the reduction in relative liver weight (%) because of the improvement in the inflammation and vascular congestion in the treated group compared to the control group after pooled fetal UCMSCs-derived sEVs preparations administration. Mendt noted the occurrence of mild inflammation in both the liver and kidneys for the treated groups. The etiology of UCMSCs-derived sEVs preparations-induced kidney inflammation in healthy rats could not be determined. More investigations on this finding using larger animal samples and different experimental designs are required to explore the findings in the future. It may be because of albumin in the pooled fetal UCMSCs-derived sEVs preparations. No association was found between albumin and kidney inflammation in the literature. However, this finding is absent in the kidneys of the rats for the assessment of the efficacy study as reported [68]. The effects of pooled fetal UCMSCs-derived sEVs preparations in the efficacy study may provide more information about where these findings may or may not be present in the kidneys of MetS rats. Surprisingly, in the lung, severe (focal) lymphoplasmacytic inflammation was observed in the control group at acute toxicity and both groups at sub-chronic toxicity. According to the histopathologist’s justification, this inflammation may be due to intrinsic factors like genetics or animal species. Pooled fetal UCMSCs-derived sEVs preparations administration may not have contributed to it.

Other internal and external factors may cause the findings and observations in the control group compared to the treated group. Since the toxicity study was conducted under the same environment and conditions, comparing the findings between the control and treated groups will provide data on whether the administration of UCMSCs-derived sEVs has any changes in the treated group. In some cases, there is improvement from the acute toxicity observation to sub-chronic toxicity, such as inflammation (lymphoplasmacytic) and vascular congestion in the liver and the absence of symptoms such as necrosis in sub-chronic toxicity compared to acute toxicity. The UCMSCs-derived sEVs may cause recovery effects from early administration (acute) to late administration (sub-chronic). The histopathologist also concluded that some observations, such as mild lymphovacular dilatation in the lymph nodes in both groups, could be a normal occurrence and had no significant effect on the organ functions. The toxicity study shall take into account gross necropsy, relative organ weight, and hematological and serum biochemistry results and not depend on the histopathological assessment alone for the overall conclusion.

For any biomedicines intended to be marketed and for obtaining the marketing authorization holder, the product developer has to demonstrate its safety and biodistribution, besides the quality and efficacy at the preclinical stage [69,70]. Despite the emerging interest and biomedicine developments in the field of EVs, only a few studies have assessed the biodistribution of EVs in vivo, which is one of the critical steps in preclinical biomedicine development [42]. Post 24 h IV injection of pooled fetal UCMSCs-derived sEVs preparations labeled with PKH26 fluorescent dye, our study found that labeled sEVs were abundantly accumulated in the spleen and liver, consistent with previous research [71,72]. Nemeth et al. have reported that Kupffer cells, hepatocytes, and liver sinusoidal epithelial cells located in the liver are responsible for the intake of EVs [73], which are modulated by scavenger receptors [74]. Moreover, depending on the method, labeling agent, and sensitivity, EVs may also accumulate and be identified in other organs and tissues [75]. If administered intravenously, specific local and systemic inflammation may be influenced by the rapid clearance of EVs by reticuloendothelial systems [76]. The spleen is highly vascularized with reticuloendothelial systems. The communication between EVs and spleen cells was expected to cause numerous physiological responses affecting local signaling activities [77]. It is also suggested that the rapid clearance of EVs from blood circulation is mediated by the action of macrophages [78,79].

The MSCs, as the parent cell and their EVs, have different perspectives on safety concerns and considerations. Phenotypic stability is the primary concern for cell-based therapies compared to EVs, where the MOA and target entity knowledge are inadequately grasped [80]. In this study, we have proven that the pooled fetal UCMSCs-derived sEVs preparations are safe regarding toxicity (acute and sub-chronic) when administered intravenously with multiple doses. This biodistribution data showed that circulating pooled fetal UCMSCs-derived sEVs preparations were detected mainly in the spleens of healthy male SD rats after 24 h of IV administration.

## 4. Material and Methods

### 4.1. Research and Animal Ethics

The study protocol was approved by the Universiti Kebangsaan Malaysia (UKM) Research Ethics Committee (JEP-2020-790), the Animal Ethics Committee of the Faculty of Medicine, Universiti Kebangsaan Malaysia (UKM) (FP/2022/YOGESWARAN/26-JAN./1218-APRIL-2022-SEPT.-2024), Medical Research and Ethics Committee (MREC), Ministry of Health, Malaysia (NMRR ID-22-00306-ZNO) and Research Ethics Committee, Military Health Services, Ministry Of Defence (PKA/JKE/28-08).

### 4.2. Safety Study Design

**Figure 9:**
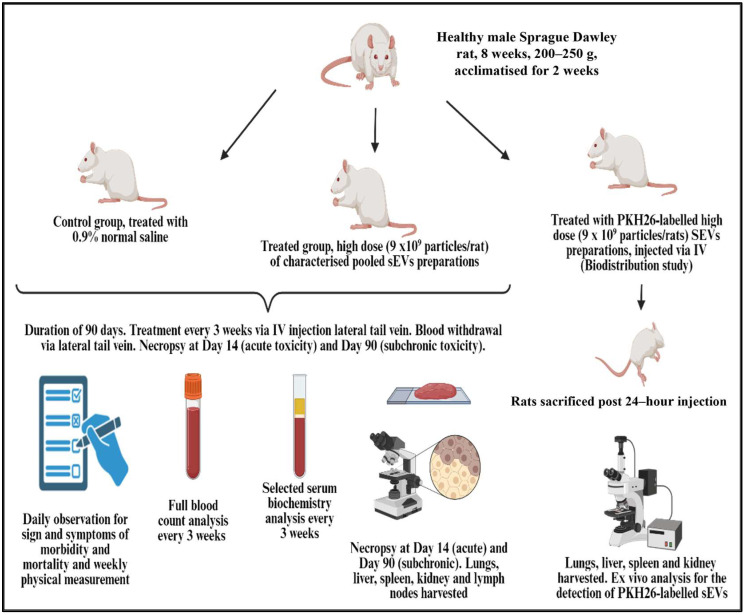
Graphical safety and biodistribution study design.

The graphical safety and biodistribution study design is shown in Figure 9.

### 4.3. Animals

Twenty-four male specific pathogen-free SD rats at 8 weeks old weighing 200–250 g were obtained from the AEU (Animal Experimental Unit), Universiti Malaysia, Kuala Lumpur. Rats were housed individually in ventilated polycarbonate cages (Allentown Inc., Allentown, NJ, USA) at a room temperature of 22 °C with 12 h light and 12 h dark cycles. All rats were acclimatized for two weeks before the experiment. The rats were fed with standard lab chow (Altromin 1314, Lage, Germany) and autoclaved tap water ad libitum.

### 4.4. Animal Treatment

Male SD rats were randomly divided into toxicity and biodistribution studies. In the toxicity study, the control group (*n* = 9) received normal saline, and the treatment group (*n* = 9) received a dose of pooled fetal UCMSCs-derived sEVs (9 × 10^9^ particles/rat) suspended in normal saline (approximately 0.3 mL) via IV injection of the lateral tail vein every three weeks. Isoflurane (Piramal Critical Care, Mumbai, India) was used as an inhaled anesthetic agent for this procedure. The rats were observed and evaluated for a study period of 90 days. During this study period, physical measurement was recorded with minimal restraint. The overnight fasting whole blood samples were collected from the rats anesthetized under isoflurane. The blood was analyzed to determine full blood count and selected serum biochemistry. These parameters were recorded at weeks 0, 3, 6, 9, and 13. At week 2, three rats were randomly selected from the treatment and control groups to determine the acute toxicity of pooled fetal UCMSCs-derived sEVs IV injection. Rats were sacrificed using pentobarbital sodium (Vetoquinol, Lure, France) overdose via the intraperitoneal route. Necropsy and harvest of organs were performed for each rat. The remaining rats (n = 6 from each group) were evaluated for sub-chronic toxicity (day 90) at the end of 13 weeks. For the biodistribution study of the fetal UCMSCs-derived sEVs, a total of six rats were involved, as explained in Section 4.6.

### 4.5. Fetal Umbilical Cord Protocol and Isolation of Small Extracellular Vesicles

The fetal part of the human umbilical cords was used to isolate fetal UCMSCs. These umbilical cords were collected from volunteer healthy-term pregnant mothers with informed consent from the Department of Obstetrics and Gynaecology at Hospital Angkatan Tentera Tuanku Mizan, Wangsa Maju, Ministry of Defence, Kuala Lumpur via either spontaneous vaginal delivery or caesarean section. Isolation and culture of fetal UCMSCs, characterization of UCMSCs, isolations of sEVs, characterizations of sEVs, and pooled sEVs preparations have been described in the previous study [81].

### 4.6. Safety Study Monitoring Parameters

#### 4.6.1. Physical Measurement and Observation

The rats were restrained minimally and measured weekly for body weight, (BW) in (g), and body length, BL (cm, from the nose to anus) using a weighing scale and measuring tape, respectively. Body mass index, BMI (g/cm^2^), was calculated by the ratio of BW to BL (squared). Food consumption was measured by weight (g) consumed per week. Fluid intake was measured as the volume (mL) of water consumed per week. Daily observation was conducted on each rat for the signs and symptoms described in Supplementary Table S1 to assess the morbidity and mortality.

#### 4.6.2. Blood Analysis

Rats were bled at weeks 0, 3, 6, and 13. Whole blood was collected in BD Vacutainer^®^ Blood Collection Tubes (BD Biosciences, San Jose, CA, USA), allowed to clot at room temperature, and centrifuged at 5000 rpm for 10 min at room temperature to obtain the serum. For the FBC, whole blood was collected in the BD Vacutainer^®^ EDTA Tubes (BD Biosciences, USA). FBC and selected serum biochemistry analysis were performed at the Chemical Pathology Laboratory in the Veterinary Laboratory Service Unit (VLSU), Universiti Putra Malaysia (UPM), Malaysia. The FBC consisted of red blood cell (RBC) count, hemoglobin (Hb) count, packed cell volume (PCV), mean corpuscular volume (MCV), mean corpuscular hemoglobin concentration (MCHC), white blood cell (WBC) count, segmented neutrophil count (NEUTRO), band neutrophil count (Band NEUTRO), lymphocyte count (LYMPH), monocyte count (MONO), eosinophil count (EOSIN), basophil count (BASO), platelet count (PLT), and plasma protein. The selected serum biochemistry test consisted of alkaline phosphatase (ALP), aspartate aminotransferase (AST), alanine aminotransferase (ALT), cholesterol (CHO), amylase (AMY), creatinine (CREAT), and lactate dehydrogenase (LDH).

#### 4.6.3. Necropsy

Necropsy evaluation was conducted twice to determine acute toxicity two weeks after treatment started and at day 90 to determine the sub-chronic toxicity by a qualified and trained veterinarian. The major metabolic organs harvested were the lungs, liver, spleen, kidneys, and lymph nodes. The gross pathological evaluation was conducted, and the relative weight of organs was calculated as the percent (%) by dividing organ weight by BW and multiplying by 100.

#### 4.6.4. Histopathological Assessment

The harvested organs were preserved in a 10% neutral buffered formalin (Chemiz, Shah Alam, Malaysia) before being embedded in paraffin wax. Sections were cut with a microtome at 5 µm, deparaffined with xylene, and stained with standard hematoxylin (Epredia^TM^, Kalamazoo, MI, USA) and eosin (Epredia^TM^, USA) staining. Three blinded histopathologists observed the stained slides under an inverted light microscope for histopathological assessment.

### 4.7. Biodistribution Study

Based on previously reported procedures [82,83], fluorescent labeling of pooled fetal UCMSCs-derived sEVs preparations was carried out using the manufacturer’s protocol. In brief, 100 µg of the pooled sEVs preparations were reacted with 4 µL PKH26 Red Fluorescent Cell Linker (Sigma Aldrich, St. Louis, MO, USA) dye for the treated group, and PBS was used for the control group. To remove the excess dye, fluorescent labeled EVs/PBS were washed 4 times using a 100-kDa filter (Microcon YM-100, Millipore, Derwood, MD, USA). These labeled dye solutions were prepared fresh before being injected intravenously into the rats via the lateral tail vein. After the acclimatization period, three rats were randomly selected and injected with 9 × 10^9^ particles of sEVs that had been labeled with PKH26, and the remaining three rats were injected with PBS-labelled PKH26 dye as a control. The rats were euthanized using the same chemical method, and their major organs, the lungs, liver, spleen, and kidneys, were harvested 24 h after the IV injection. These organs were stored in PBS solution at −80 °C. Cryo sectioning was performed using an optimal cutting temperature compound (Tissue-Tek^®^, Sakura, Japan), and the sample was sectioned at 3 µm. This sample was counterstained with Hoechst dye (Sigma-Aldrich, USA) and incubated for 30 min at room temperature. The slide was viewed under an inverted fluorescence microscope (Nixon Eclipse Ti, Tokyo, Japan), and the images were captured.

### 4.8. Statistical Analysis

Statistical analysis was performed using GraphPad Prism version 9.0.0 (GraphPad Software, San Diego, CA, USA). All quantitative variables were presented as mean ± standard error mean (SEM). Comparisons between treated and control groups for physical measurement, selected serum biochemistry FBC, and biodistribution tests were conducted through mixed-effects repeated measures two-way analysis of variance (ANOVA) with Geisser–Greenhouse correction. The time-point pairwise intergroup analysis was calculated using Sidak’s post-hoc test, while the intra-group analysis used Tukey’s post-hoc test. The multiple comparisons were indicated by the least significant difference when there was a significant difference between the groups. The end-point comparison of the relative weight of the organ was calculated using an independent *t*-test. A difference at *p* ≤ 0.05 was considered statistically significant.

## 5. Limitations

This study presents several limitations that should be considered when interpreting the results. First, all experiments were conducted using only male rats, which limits the generalizability of the findings across sexes, as hormonal differences in females may influence the biodistribution and toxicity of sEVs. Second, the sample size for the acute toxicity and biodistribution studies was relatively small, potentially reducing the statistical power to detect subtle effects. Third, biodistribution analysis was limited to a single time point at 24 h post-injection. While sEVs were observed in the liver, spleen, and lungs, the long-term retention and clearance profile of these vesicles remains unknown. Further time-course studies are needed to fully elucidate the pharmacokinetics and tissue dynamics of sEVs. Additionally, mild focal lymphocytic infiltrates were observed in the lungs of both treated and control groups. These findings may reflect incidental or strain-related background pathology commonly reported in rodents, rather than treatment-related effects. The presence of albumin in the UCMSCs-derived sEVs preparation [81] may induce inflammation in the treated group. This finding is only observed in the healthy rats and is absent in the disease animal model [68]. However, no significant differences were noted in relative kidney weight and gross necropsy evaluation. Besides that, no significant differences were observed in the serum creatinine of the rats between the two groups. No spare serum is available for further renal biochemistry evaluation, such as potassium and sodium levels.

## 6. Conclusions

In conclusion, in this study, we evaluated the safety of the pooled fetal UCMSCs-derived sEVs administered intravenously in healthy male SD rats. The evaluated parameters were daily observations for signs and symptoms of morbidity and mortality, physical measurements (BW, BL, BMI, food consumption, and fluid intake), hematological assessments (RBC, Hb, PCV, MCV, MCHC, WBC, NEUTRO, Band NEUTRO, LYMPH, MONO, EOSIN, BASO, PLT and plasma protein), biochemical assessments (ALP, AST, ALT, CHO, AMY, CREAT and LDH), gross necropsy, relative organ weight (%), and histopathological assessment of the lungs, liver, spleen, kidney, and lymph nodes. Multiple doses (9 × 10^9^ particles/rat, every three weeks) via IV injection of the pooled fetal UCMSCs-derived sEVs preparations did not produce any apparent adverse effects on the rats in acute and sub-chronic toxicity assessment. No death occurred throughout this study, and none of the rats had any signs or symptoms of morbidity and mortality. Significant results were noted for food consumption and fluid intake in the physical measurement between both groups, but these findings have no contribution to the toxicity of the rats. For the hematological and biochemical parameters, significant differences at week 0 were observed for both PCV and ALP before the start of the treatment, and no significant differences were observed thereafter. Therefore, these findings were not associated with the toxicity of sEVs administration. Plasma proteins were significantly lowered at week 6 for the treated group compared to the control group. Based on the histopathology assessment, pooled fetal UCMSCs-derived sEVs preparations could improve liver recovery following inflammation and vascular congestion of unknown cause, probably through their anti-inflammation and immunomodulatory properties. However, cautious and further investigation is needed to assess the focal mild inflammation in the kidney after administering the pooled fetal UCMSCs-derived sEVs. Overall, this study has been achieved in evaluating the safety aspects of human fetal UCMSCs-derived sEVs preparations in healthy male SD rats, and these results showed that this dosing regimen is comparatively safe in healthy male SD rats. Biodistribution of the pooled fetal UCMSCs-derived sEVs preparations showed mostly accumulated in the spleen, followed by the lungs, liver, and the least in the kidney. Overall, this study provides a safety and biodistribution profile as essential groundwork for the future application of EVs as nano biomedicines in preclinical efficacy studies and clinical trials.

## Figures and Tables

**Table 1:** Gross necropsy examination of the harvested organ.

Organ	Day 14 (Acute Toxicity)	Day 90 (Sub-Chronic Toxicity)
Lungs	Normal	Normal
Liver	Normal	Mottled edge appearance control (n = 3) and treated (n = 3)
Spleen	Normal	Blunt edge appearance control (n = 1) and treated (n = 1)
Kidney	Normal	Normal
Lymph nodes	NP	Reddish appearance treated (n = 3)

NP: not performed. n = 3 from each control and treated group on day 14. n = 6 from each control and treated group on day 9.

**Table 2:** Summary of histopathology assessment for lungs.

Assessment Parameter	Acute (D14) Toxicity		Sub-Chronic (D90) Toxicity	
	Control	Treated	Control	Treated
Necrosis	NIL	NIL	NIL	NIL
Pulmonary oedema	NIL	NIL	NIL	NIL
Inflammation (lymphoplasmacytic)	Severe (focal)	NIL	Severe (focal)	Severe (focal)
Inflammation (neutrophils)	NIL	NIL	NIL	NIL
Haemorrhage	NIL	NIL	Mild	NIL

**Table 3:** Summary of histopathology assessment for liver.

Assessment Parameter	Acute (D14) Toxicity		Sub-Chronic (D90) Toxicity	
	Control	Treated	Control	Treated
Necrosis	Mild	Mild	NIL	NIL
Apoptosis	NIL	Mild	NIL	NIL
Inflammation (lymphoplasmacytic)	Mild	Mild to moderate	Mild	Mild
Inflammation (neutrophils)	NIL	NIL	NIL	NIL
Vascular congestion	Mild	Moderate	Moderate	Mild
Haemorrhage	NIL	NIL	NIL	NIL

**Table 4:** Summary of histopathology assessment for spleen.

Assessment Parameter	Acute (D14) Toxicity		Sub-Chronic (D90) Toxicity	
	Control	Treated	Control	Treated
Necrosis	NIL	NIL	NIL	NIL
Apoptosis	NIL	NIL	NIL	NIL
Largest size of lymphoid follicles (mm)	0.5	1.0	2.0	1.5
Giant cells	NIL	NIL	NIL	NIL

**Table 5:** Summary of histopathology assessment for kidney.

Assessment Parameter	Acute (D14) Toxicity		Sub-Chronic (D90) Toxicity	
	Control	Treated	Control	Treated
Necrosis	NIL	NIL	NIL	NIL
Apoptosis	NIL	NIL	NIL	NIL
Inflammation (lymphoplasmacytic)	NIL	NIL	NIL	Moderate (focal)
Inflammation (neutrophils)	NIL	NIL	NIL	NIL
Tubular changes (dilation)	NIL	Mild	Mild	Mild

**Table 6:** Summary of histopathology assessment for lymph nodes.

Assessment Parameter	Sub-Chronic (D90) Toxicity	
	Control	Treated
Necrosis	NIL	NIL
Apoptosis	NIL	NIL
Lymphovacular dilatation	Mild	Mild
Lymphoid follicles diameter (mm)	0.4	0.4
Giant cells	NIL	NIL
Granuloma	NIL	NIL

n = 3 from each control and treated group at day 14. n = 6 from each control and treated group at day 90.

## Data Availability

The data supporting this study’s findings are available from the corresponding author upon reasonable request.

## Supplementary Materials

The following supporting information can be downloaded at https://www.mdpi.com/article/10.3390/ijms26146806/s1.
