# Supplementary material for: Safety Evaluation and Biodistribution of Fetal Umbilical Cord Mesenchymal Stem Cells-Derived Small Extracellular Vesicles in Sprague Dawley Rats
Source: Int J Mol Sci. 2025 Jul 16;26(14):6806. doi: 10.3390/ijms26146806 (PMC12295939; doi:10.3390/ijms26146806)
Supplement: Supplementary file 1 [file ijms-26-06806-s001.zip › ijms-3685042-supplementary.pdf]

Supplementary Table

Table S1: Morbidity and mortality of rats throughout the study (daily observation)

Symptoms	Study Period					
	Acclimatisation	Week 0	Week 2	Week 4	Week 8	Week 13
Anorexia, weight loss, and/or dehydration (related to food and water intake)	-	-	-	-	-	-
Dyspnea (labored breathing, hyperventilation, abdominal distension)	-	-	-	-	-	-
Prolonged hypothermia or hyperthermia (palpable temperature)	-	-	-	-	-	-
Stress and/or poor grooming (rough or stained coat and porphyrin built around nose and eyes)	-	-	-	-	-	-
Lethargy, hunched posture, and inability to rise or ambulate	-	-	-	-	-	-
Poor reflex or irresponsiveness to external stimuli	-	-	-	-	-	-
Tumor growth	-	-	-	-	-	-

Symbols: (-) absent of the symptoms.
